# Medical technologies, time, and the good life

**DOI:** 10.1007/s40656-022-00504-z

**Published:** 2022-06-09

**Authors:** Claudia Bozzaro

**Affiliations:** Institute for experimental Medicine, Department of Medical Ethics, Christian-Albrecht’s-University Kiel, Kiel, Germany

**Keywords:** Finitude, Temporality, Good life, Social egg freezing, Anti-aging, Enhancement

## Abstract

Against the backdrop of emerging medical technologies that promise transgression of temporal limits, this paper aims to show the importance that an individual lifetime’s finitude and fugacity have for the question of the good life. The paper’s first section examines how the passing of an individual’s finite lifetime can be experienced negatively, and thus cause “suffering from the passing of time.” The second section is based on a sociological analysis within the conceptual framework of individualization and capitalism, which characterizes many modern individualized and consumerist societies and explains how the described problem of time’s passage is particularly relevant today. The paper then proceeds to show and discuss how individuals employ various, primarily medical, enhancement-technologies like social egg freezing, anti-aging-medicine and physical- and neuro-enhancement in an attempt to overcome time’s passing. Finally, the paper seeks to explain why such attempts fail and, moreover, why it is exactly the awareness of time’s passing that can constitute a prerequisite for a good life.

## Introduction

Time is elusive, yet simultaneously, time significantly determines our way of thinking, our actions, and our lives. We must bow to time’s biological rhythms, as these determine our bodily existence. We take up other people’s time while we concurrently adjust our own temporal rhythms to those of other people and society. We reckon with time; we organize and manage it. We try to find our place within history, and yet we must cope with the fact that at some point history will go on without us. Above all, though, we are time ourselves, namely in the sense that the fundamental structure of our lives is a temporal one. Our life is delimited by our own birth and death, while our experiences are constituted through our consciousness of time. The passing of our finite lifetime essentially determines the way we approach and plan our life. The passing by of an individual finite lifetime is the form of temporal experience to be addressed within the framework of this article. To put it more precisely: This paper explores the meaning the temporality of human life has when it comes to confronting the issue of *the good life* - specifically its finitude and fugacity. According to Aristotle, who first coined the term good life, there is an end or purpose to all the actions that we perform. Further, we desire this purpose - or highest human good - for its own sake, with all other things and aspects of life being desired on its account (Anscombe, 1958; MacIntyre, 2013). In modern words, it may be translated with happiness (in a non-utilitarian or hedonistic sense) or an accomplished life, and implies the development of character traits, namely *virtues* or *excellences*, which shape the individual toward this end.

This paper starts with the assumption that people living in individualized and consumerist societies have a plethora of diverging life choices. The finite time they have at their disposal will never be enough to realize all these options. This then poses the question as to how they can live a good life in the face of their finiteness.

The development of several medical technologies like social egg freezing, anti-aging-medicine, and physical-/neuro-enhancement can be interpreted as a response to this problem. Social egg freezing refers to a reproductive medical procedure by which unfertilized oocytes can be stored for a long period of time. At a later point in time, which can also be beyond a woman’s menopause, a woman’s thawed and in vitro fertilized eggs can be transferred and lead to a pregnancy. This measure was developed for oncological patients, but is now requested more and more frequently by women who postpone childbearing for social reasons and who aim to ´preserve´ their fertility (Alteri et al., 2019; Waldby, 2015).

A number of very heterogeneous medical interventions - from the use of nutritional supplements to surgical cosmetic procedures - can be subsumed under the term *anti-aging medicine*. While some representatives see the goal of anti-aging medicine as a slowing down of the aging process and in the so-called compressed morbidity (Fries, 2005), others aim for the abolition of the aging process itself (Binstock, 2003; De Grey & Rae, 2007).

Physical and neuroenhancement methods refer to an equally broad range of different measures that ultimately aim to improve physical and cognitive abilities. The aim is not to restore a ´healthy´ or ´normal´ state, but to increase, to improve beyond the healthy and normal state. For this purpose, drugs are often used which had a different primary development goal. An example of this is when adults take Ritalin - a well-known drug for the treatment of attention deficit/hyperactivity disorder (ADHD) in children. Adult users, however, turn to Ritalin because it increases their ability to concentrate (Allhoff et al., 2011; Schöne-Seifert & Talbot, 2010).

At a first glance, the assumption that medical technologies might have something to do with time is not evident. However, the connection to time becomes obvious when the figurative and linguistic metaphors used in connection with these technologies are examined more closely. This becomes most obvious in the context of social egg freezing where the image of the ´biological clock´ plays a major role (Amir, 2007). The biological clock marks the time a woman has left before her fertility reserves are exhausted. The closer a woman gets to the critical age at which, at least statistically, her reproductive possibilities decrease, the more clearly she perceives the ´ticking of her biological clock´. Social egg freezing, as will be shown in more depth later, intends to remove this disturbing ticking and to bring the timeframe of reproduction into the woman’s power.

The slogan ´Stop the clock!´ is also frequently used in the context of anti-aging medicine (Klatz, 2009). Here, however, a different clock is meant, namely the one that measures the passing of the individual lifetime. The offer of anti-aging medicine is not only aimed at slowing down or stopping the passing of time, but time is even supposed to be reversed and the hands of time turned back, as it were. How is this supposed to work? The trick is to distinguish chronological age, which is determined by the date of birth, from biological age. Biological age measures, so to speak, the ´real age´ condition of the body. This depends not only on genetic, but also on life-style factors that are quite modifiable. Some representatives of anti-aging medicine go so far as to promise rejuvenation in the old tradition of the fountain of youth (Ullis, 2012).

In connection with physical- and neuro-enhancement, another temporal aspect is in the foreground, namely speed. These technologies are often presented as weapons in a battle, a competition against time, in which time is not to be stopped, but simply caught up with and preferably overtaken. In relation to physical enhancement in sports, this is meant quite literally. In some sports, the ultimate goal is to break record speeds. This aspect also plays an increasingly important role in the world of work and in private life, where the use of performance-enhancing substances is intended to enable people to become ever better, effective, and efficient, which always means faster and more time-effective (Lad & Harrison, 2012).

This paper starts from the assumption that the use of the mentioned biomedical technologies indicates that at least some people nowadays have a rather problematic relationship with time, which leads to them taking action against it. In a first step, (1) this assumption will be illustrated by the description of the problem of the passage of time and the way the mentioned biomedical technologies are supposed to answer this problem. Then (2) the limits and problems of these attempts are discussed. The final part of the paper aims to argue that the awareness of the finitude and fugacity of one’s own lifetime can be a prerequisite for a good life, while attempts to gain more life through medical technologies may ultimately prove deceptive. This argument will be developed on the basis of thoughts inspired by the philosopher Søren Kierkegaard on the meaning of the finitude and fugacity of time for the question of the good life are discussed. Kierkegaard was chosen because he is the founder of existentialism, a philosophical tradition that is mainly concerned with individuals’ struggle in the face of the finitude and contingency of humans’ existence.

## The human being: a ´time famine creature´

Following Arnold Gehlen, the German philosopher Odo Marquard (Marquard et al., 1995) once described the human being as a ´time famine creature´ - in German, ´*Zeitmängelwesen.´* This means, due to their finite nature, human beings suffer from a chronic lack of time. This problem can be understood as a *conditio humana* (Schües, 2014) The finiteness of human life has always been a challenge that has been dealt with by corresponding myths, narratives, and religious and metaphysical attempts at explanation. In the context of individualized and consumerist societies, this problem takes on a new relevance and urgency due to some characteristics of those societies and of their anthropological presuppositions.

Following sociological analyses like those proposed by Ulrich Beck and Elisabeth Beck-Gernsheim (Beck & Beck-Gernsheim, 2002), as well as Zygmut Bauman (Bauman, 2013), individualized and consumerist societies can be characterized by individualism and capitalism. People are understood to be autonomous, independent individuals who are – or at least should try to be – free from the constraints of history, society, and other people. According to this assumption, individuals have their private and authentic preferences, values, attitudes, and life goals. Individualized and consumerist societies are supposed to give people the freedom to discover and develop their individual and authentic preferences and values and to live their own life in order to achieve their personal life goals. Accordingly, individual freedom, understood in the sense of self-determination, is accorded the highest value. As psychologists like Barry Schwarz and Nathan Cheek (Schwartz & Cheek, 2017) emphasize, individuals tend to consider their own life as the result of their own choices and actions, rather than accidents, luck or contingency. The assumption of an individualized and consumerist rationality is that individuals are free to choose and are responsible for giving their lives meaning and to finding ways of self-realization. Contemporary individualized and consumerist societies are mainly dominated by the economic principles of capitalism. Individuals are confronted with an exponential increase in the range of lifestyle options and possibilities for self-realization. Many people, at least in the middle and upper classes, find themselves confronted with unconstrained options when it comes to where they live, what they study, what kind of job they want, what kind of intimate relationships they will enter, and so on.

For individuals living individualized and consumerist societies, situations can evolve which cause them to ´suffer from the passing of time´ (Bozzaro, 2014) and which motivates the use of biomedical-technologies in order to overcome this suffering. In the following, an analysis of what such a suffering of the passing time can consist of, and which possible answers medical technologies offer, is performed.

### Suffering from the passing of time: “freezing time” through social egg freezing

Due to its finitude and its fugacity, time can sometimes painfully pressure individuals to make decisions at a certain point in their lifetime - even if they do not want to or feel unable to make them at that moment. An example of this kind of suffering due to temporality is being experienced more and more often by women (Daly & Bewley, 2013). For centuries, childbearing was a woman’s main duty and motherhood was her self-evident social role. However, since the 1970s, the availability of effective contraceptive methods has served as a stepping stone for broader female emancipation, leading a growing number of women to take advantage of educational, employment, and career opportunities and become increasingly independent – also from an economic perspective (Callahan, 2009; Coontz, 2004). Since education and starting a career are time consuming and not always compatible with child care, there has been a massive delay in childbearing, especially among highly educated women (Mills et al., 2011). The trend towards delaying parenthood can be observed in the vast majority of OECD countries. Between 1970 and 2019, the mean age of women giving birth increased by between two and five years in most OECD countries, with a mean age at first birth of 30 or above (OECD Family Database www.oecd.org/social/family/database, accessed September 17, 2021).

The assumption of an individualized and consumerist rationale is that individuals are given freedom of choice and therefore responsible for the consequences of the choices and decisions they freely make (Hayek, 2012). Individualized and consumerist ideology is distinct from other ideologies that also accentuate individual freedom of choice and responsibility, such as in the philosophy of the Enlightenment (e.g. Immanuel Kant), as freedom is seen only as an expression of personal preferences and individual values. As critics like Michael Sandel and Charles Taylor observe, neo-liberal ideology does not take into consideration that an individual’s freedom is often coerced by social constraints. This is especially true in consumerist societies where personal preferences and values are often deeply influenced by the offers of the market and advertising (Sandel, 1982; Taylor, 1992). This ideology influences how childbearing and motherhood are seen and approached.

Women fortunately have more freedom to plan their lives according to their wishes and expectations (Goldin, 2006). Yet at the same time, they face new societal expectations. As Elisabeth Beck-Gernsheim (Beck-Gernsheim, 1988) points out, and as analysis from media-discourses confirm (Budds et al., 2013), a woman nowadays does not just have the option, but rather the obligation, to plan her life according to rational and responsible considerations. Women want, need, and are expected to graduate and to have successful careers. Since divorce rates have increased and marriage no longer serves as an institution that safeguards women without income, women also need to be financially independent. In order to achieve all this, women must organize their lives in a conscious, “rational” way, which includes using the contraceptive methods offered by reproductive medicine to schedule their reproduction. As Beck-Gernsheim shows, young women who do not use contraception and thus “risk” getting pregnant are “endangering” their education and careers and may now be accused of acting unreasonably and irrationally (Beck-Gernsheim, 1988; Budds et al., 2013). At the same time, as some feminist scholars claim, modern societies are still “implicitly pronatalist” (Smajdor, 2009). It can be seen that great importance is also placed on women having children. Since it is well known that late pregnancies bear more risks, women are also expected to choose the “biologically optimal” time to conceive (Carroll & Kroløkke, 2018). Considering these points, the current social expectation of women is: To be a “modern” woman, they should pursue education, employment, and financial independence, but at the same time they should not forget to settle down in a stable relationship to have children at the “right” time and be a “responsible” mother, too. Women are expected to ideally combine social and biological time constraints.

The timing of pregnancy is not always a straightforward choice that depends solely on a woman’s life plans and her priorities. As research has shown, one of the most important prerequisites people consider for parenthood is a stable relationship (Baldwin, 2019; Eriksson et al., 2012; Hodes-Wertz et al., 2013; Stoop et al., 2015). Therefore, before a woman can realize her desire for a child, she must first find a suitable partner. For an increasing number of women, however, the search for ´Mr. Right´ turns out to be tedious and time-consuming. As the Israeli Sociologist Eva Illouz (Illouz, 2012) illustrates in her well-known book *Why Love Hurts*, having a child is often seen as the culmination of a stable, romantic relationship in individualized and consumerist societies. Modern love is characterized by liberalization, which means that people are free to choose their partners in accordance with personal preferences. Increased mobility and new technologies, such as internet-dating, make it possible to interact with more and more people from all around the world, meaning the field of potential partners has grown. But at the same time, as Illouz notes, the uncertainty about how to make ´the right´ or ´good’ choice in the face of so many possibilities have grown, too. This has led to a high number of young adults seemingly no longer able to make a suitable choice for a stable relationship at all. For women who desire motherhood, a painful situation can result since their “time-window” for conceiving naturally narrows due to biological constraints. Therefore, they may end up in a situation where they feel pressured by their so-called biological clock, a time-metaphor that is omnipresent in the discourse on social egg freezing (Amir, 2007). This can lead to a woman wanting to ´freeze time´, and consequently stop the pressure time is putting on her (Baldwin et al., 2019). With egg freezing, a woman can entertain, at least theoretically, the possibility of becoming pregnant at a later point in time, even after the ´natural border´ of menopause. The egg freezing procedure consequently promises to liberate women from their biological clock by shifting a boundary temporality placed on their reproductive possibilities.

### Suffering from the passing of time: “stop and reverse time” through anti-aging-medicine

The experience women have had regarding their limited reproductive possibilities can be interpreted as a peculiar experience of finitude and fugacity. The suffering which can arise is caused by being confronted with an important life choice – having children – that simply will not be available after a certain point in time. Not only women’s reproductive possibilities are finite and ephemeral, but also the lives of all human beings. This *conditio humana*, however, is a special challenge in individualized and consumerist societies, where an individual´s expectation of self-fulfillment and happiness become higher and higher in proportion to the possibilities that a whole industry is producing for its consumers (Bauman, 2013). Often, one becomes aware of the finitude and the irreversibility of one’s own life through the experience of aging. In one’s youth, the future may seem endless and therefore, endless opportunities of self-fulfillment and life-projects seem possible. Through aging, one becomes more and more aware of time passing and the possibilities and experiences that could have been. Throughout a lifetime, one can only choose a limited number of projects and experiences, meaning others must be excluded. Considering this, becoming aware of one’s own aging can lead to making oneself painfully notice the time constraints on an individuals’ expectations and wishes.

There are at least two ways people can react to this problem with anti-aging medicine. The first is, analogous to the answer given through social egg freezing: trying to ´stop time´. Often, particularly physical changes create awareness of aging, in both oneself and others. Gray hair and wrinkles that appear on the face are visible signs of a temporal process that all people are subject to. Anti-aging treatments, such as cosmetic surgery, are supposed to eliminate these visible signs of aging to maintain a more youthful appearance. The second way is to ´reverse aging´. As mentioned in the introduction, for some proponents of anti-aging medicine, the aim is not only to stop the aging process, but to reverse the passing of time. Here the distinction between biological and chronological age becomes crucial. While the biographical, or chronological age, is calculated according to the date of birth, the biological age determines the ´actual´ state of a person’s bodily and mental fitness, and is measured by various biomarkers. Since, in addition to epigenetic factors, these values are supposed to depend mostly on one´s lifestyle, biological age can be influenced (Abbott, 2019). Prevention and comprehensive anti-aging treatments, such as a ketone based diet that seemingly has a rejuvenating effect on brain activities (Weistuch et al., 2020), can not only help to avoid premature aging, but even rejuvenate one’s own biological age. Stopping and reversing the aging process to maintain youth can be interpreted as an answer in the face of a suffering caused by finite time passing by. Maintaining youth means to maintain an open future, with open windows of opportunity. (Bozzaro, 2014).

### Suffering from the passing of time: “acceleration” through enhancement

Individualized and consumerist societies have been described as, among other things, performance-oriented societies that are mainly dominated by the economic principle of capitalism (Rosa, 2013; Stein, 2018). This also has consequences for the perception of time, because “time is money,“ meaning that time should be used in an economically effective way. A common way to use time optimally is by accelerating activities and processes. As sociologists like Paul Virilio (Virilio, 1986) and Hartmut Rosa (Rosa, 2013) have shown, modern societies distinguish themselves through an enormous acceleration of all of time’s rhythms. In both the world of work and the private sphere, individuals must make an optimal allocation of their time resources to fulfil their own and others’ expectations, and to attain all possibilities of their self-realization. Individuals who want to hold on to their career aspirations and simultaneously not miss out on life or on leisure activities must compensate for the brevity of their lifetime by speeding through life. One way to increase tempo has come through the development of new ´time saving´ technologies, such as washing machines: There is no doubt that cleaning clothes in the washing machine is much faster and easier than if one washed them by hand. While the machine cleans the laundry, one can do something else.

Another way to gain time is to improve one’s own physical and cognitive abilities to do things more efficiently. Using doping, and especially through cognitive or neuro-enhancement, one can expect to boost one’s own physical and cognitive capacities to do things better and faster. ´Optimizing´ the need for sleep, or the ability to concentrate, one can not only do things better and more effectively, but also faster. Empirical studies have shown that, for example, the (mis)-use of Ritalin or methylphenidate as a neuro- or cognitive enhancing substance has increased (McDermott et al., 2021). Ritalin is an amphetamine drug type that is typically prescribed as therapy to individuals with ADHD. In recent years, the use of Ritalin among healthy adults, such as students, to enhance cognitive functioning has been increasing. Students mention the improvement of academic performance as one main reason for the use of this drug (DuPont et al., 2008; Peterkin et al., 2011).

## The ambivalent nature of medical technologies as responses to the suffering of time passing by

People suffering from time passing may attempt to master their problem through boundary shifting with social egg freezing, repression (*Verdrängung*) of the aging process, through anti-aging medicine, or through physical- and neuro-enhancement. The nature of these strategies to compensate for time is ambivalent. They can lead to gains in time, but also foster undesirable side effects.

The attempt to break through the boundaries of time with social egg freezing does, at minimum, offer a momentary liberation from the pressure felt to make a definite choice immediately. In this regard the procedure can be seen as a way to empower women and to promote gender equality (Goold & Savulescu, 2009; Weber-Guskar, 2018). As empirical studies have shown, women who perform social-egg-freezing consider this option as a way to gain control over their reproduction and don´t regret their decision in favor of the treatment (Stoop et al., 2015).

Women may believe they have ´bought a little biological time´ and the costs and small risks associated with the procedure may well be worth taking for that sense of empowerment. However, it is vital that women, especially if they are over 35, are made aware that their frozen eggs do not represent an insurance policy against age-related infertility. While boundary shifting can lessen the pressure of time, it cannot solve the basic challenge: That time passes by and that some life choices must be made under the given social conditions and at a point in time when certain circumstances are given. For a few women social egg freezing may be a good solution, but it does not solve the structural societal problems that lead to many women to postponing motherhood and feeling stressed by their biological clock. There is reason to fear that if an increasing number of women do decide to make use of the technology, the structural, societal constraints might never be addressed (Shkedi-Rafid & Hashiloni-Dolev, 2011). The result would be that in future, women would be expected to use social egg freezing to further adapt to the social constraints. Additionally, social egg freezing does not solve - and indeed may very well tend to reinforce - the psychological reasons that have led to a lot of young adults seemingly being no longer able to settle down in a stable relationship. When making a decision is no longer urgent, the danger of living “a life on perpetual postponement” can arise (Lockwood, 2011; Perrier, 2013). This means living a life in which decisions are put off from one day until the next until eventually, they might never be actively taken. The first empirical studies on social egg freezing show that only very few women who have undergone the procedure for social reasons ever come back and ´make use of´ their frozen eggs (Hodes-Wertz et al., 2013; Myers et al., 2015; Stoop et al., 2015). In a recent study from Jones at al. 2020 only 3,4% of women who stored their oocytes used them (Jones et al., 2020). Of course, some of these women will have gone on to become pregnant in ´natural´ ways. However, this data can also be interpreted differently: For most of these women, the social and psychological constraints have probably not changed. Gaining a little bit more time is not the same thing as solving these issues (Bozzaro, 2018).

The repression of the fact that our own lifetime is passing, which anti-aging medicine abets through its means of masking and concealing the signs of aging, is also not a satisfactory solution. This is because it is, at its very core, based on an illusion. At least until now, the passing of time *alias* the aging process cannot really be arrested or reversed. It is true that the mean life expectancy has grown and that more and more people reach an older age in a healthy condition. This is undoubtedly an advance that owes much to medical and economic developments. If anti-ageing medicine is understood as a way of shaping good ageing, it can certainly make an important contribution to more and more people reaching a healthy and good old age. However, this is not the same as to promise that the aging process can be arrested or reversed. The youthful and attractive appearance, and the health and fitness that aging people can obtain through anti-aging treatments and regimes is still different from the beauty, attractiveness, and health that the young enjoy. Older people must always fight against aging to maintain their youthful appearance. The youthful mask must constantly be touched up and done so ever more frequently, because whilst time may have been concealed, it has in no way been suspended. The individual who wants to suppress time risks to end up becoming the slave of passing time in that they are always trying to get ahead of time in order to sustain the illusion (Bozzaro, 2014). To bring about this standstill of appearance - and maintain it - the aging person themselves can no longer stand still. The individual is in danger of falling into a spiral that constantly accelerates. Once in it, there is no escaping this spiral as long as a person wants to maintain the illusion of eternal youth. The paradox and actual danger of this effort of repression ultimately stems from this: Individuals desperately trying to stop time eventually find they have squandered away the lifetime that is actually present in exchange. In a way, they subject themselves to the dominion of time, as in their attempt to ´trick time´, they take on its restless movement. This becomes clear, for example, when we use Botox and can’t stop, because then the youthful appearance crumbles right away. We have to spend more and more time performing procedures, which paradoxically leads to a constant preoccupation with our aging process.

As mentioned above, anti-aging-medicine is not a homogeneous field. While some only aim to postpone aging, others explicitly aim to avoid death by aging, and wish to gain the possibility of reaching the age of 200 or even 500 years. Regarding the latter, a further remark is needed: The wish for immortality has always been present in the history of humankind. It was there when *The Epic of Gilgamesh* was written, and at the time when Lucas Cranach the Elder painted his famous *The Fountain of Youth*. In many nations, medical and technological progress, such as the development of antibiotic therapy and vaccines, has helped to achieve an average life expectancy that is already twice that of our ancestors, just in the last century. None would question that this is a great, even amazing development. But the fact is: Despite the advancements already made, humankind still wants to gain even more time. This demonstrates that, in the end, we apparently remain unsatisfied. Perhaps one might consider that gaining more and more time is not what really matters.

The attempts at using physical-/neuro-enhancement and other technologies to gain time by speeding up the processes involved in our work and actions have certainly been successful when it comes to some activities. Writing a text on a computer is definitely faster than with quill and ink, whereas taking Ritalin can help one to prepare better and faster for an exam. Nevertheless, it does not appear that such technologies decelerate the rhythms of an individual lifetime, or those of society. Rather, the opposite seems true. The problem in the logic of modern capitalism and societal acceleration is that these become more and more self-reinforcing. As individuals evolve to keep up with societal acceleration, the number of tasks expected of them increases - as do the ´must-do´ experiences they should not miss out on. For example, although the use of Ritalin makes exam preparation go faster, what students are expected to accomplish has changed, too. Students are supposed to graduate as soon as possible to then get a good job. The time “saved” by acceleration, by being able to prepare faster for an exam, is immediately ´consumed´ as the next exam is waiting. The problem of the economic principle and acceleration is that they have no end goal but tend to become an end in and of themselves.

It is evident that the strategy of increasing acceleration and maximization is effective from the perspective of an economic system that tends just to gain more and more from an individual’s work. But at least from the perspective of the individual “caught up” in the system, this strategy can turn out to be dangerous. Contemporary individuals must rush through their lives to gain more time and meet more and more expectations up to the point of exhaustion, or until they are completely spent and collapse. The spread of ´epochal illnesses,´ such as depression or burnout, could definitely be interpreted as the downside to this acceleration (Ehrenberg, 2009; Han, 2015).

## The passing of time as a chance to pose the question of a good life

As we have seen, the problem with the finitude and fugacity of time in individualized and consumerist societies becomes a problem of choice. It is a problem of an overload of possibilities of choice in the face of a limited time window in which to realize them. Thus, the anguish of missing out on the most important, or ´the best´ is even becoming a basic feeling in the lives of contemporary individuals. The suffering from the passing of time is due to several factors: the fear of making the wrong choice; the frustration of having to make a choice at all; not being allowed to learn through trial and error; not being able to do all the things we want due to a lack of time; and the overwhelming number of possibilities there are to choose from. Concurrently, the passing of time also makes reaching a decision urgent, and among other things, painful. Since time cannot be turned back, decisions cannot simply be undone. Moreover, missed opportunities often do not return. This, as Thomas Fuchs (Fuchs, 2013) demonstrated, is exactly the ´unlived life´ that can become the cause of new suffering in the form of failure and regret.

We have seen that attempts to use medical technology to buy more time and alleviate decision-making pressure have met with only limited success and cannot be seen as a means of achieving a good life. Is there a different way to deal with the finitude and fugacity of one’s lifetime to reach a good life? To answer this question, we need to firstly clarify this: When it comes to making choices about our lives, the real challenge is that we cannot foresee the consequences and impact our decisions will have in the future. Further, we also do not have an overview of, and cannot predict, the entire scope of future scenarios and events in our lives. We must design our lives blindly and we often can only judge in retrospect what consequences a decision has had, and the entirety of the worth of what we have experienced. The future-oriented perspective of our own life changes when we reach old age. Old age is often seen as a phase of life where, due to typical age-related afflictions and constraints, we can no longer actively participate in life. Through the distance that we gain - or are forced to take - towards active life, we can gain a more comprehensive view of our life’s course (Rentsch, 2016). When we have this distanced view of our own life story, we can begin to realize what was really right and important, and also what was wrong or irrelevant. Admittedly, such insights seem useless for one’s own existence if one only derives them at the point when one’s own individual life that has been crafted is ending - and it is no longer possible to live that particular life differently. But for those still young enough to shape their own way of life, it could be important to know how to anticipate this knowledge. Could this be possible?

To answer this question, some of the Danish philosopher Søren Kierkegaard’s thoughts are relevant. Kierkegaard can be considered the founder of existentialism, a philosophical tradition concerned with the individual and his attitude and responsibility towards his own life. He was one of the first philosophers to address existential states of anxiety or despair. He dealt extensively with the existential challenges of *Either/Or* in face of the finitude of human life (Kierkegaard, 2004) and inspired many other existentialist philosophers like Martin Heidegger and Jean-Paul Sartre. In doing so, Kierkegaard developed some ideas that are relevant for the question of the good life lived under the conditions of late modern and contemporary societies. In his short essay *At a Graveside* (Kierkegaard, 2000), Kierkegaard invites the individual to take an earnest attitude towards their own temporality. By this, he means each individual must “rehearse thinking about their own death” - about their own finitude. In the passage *The Decisiveness of Death*, a subsection of his book *Three Discourses on Imagined Occasions*, Kierkegaard introduces three important aspects relevant to his understanding of the concept of time and the perception of the role of death, or more specifically, of the awareness of finitude. First, he notes that the passing of time cannot be arrested and that all human efforts to bring time to a standstill are destined to fail. Accordingly, he would also understand the attempts described above to influence the passing of time through medical technology as useless. The second aspect is that time not only passes relentlessly, but it also sweeps the individual away with it. This means, as already noted above, that time forces one to follow its course in that the individual must continuously handle what comes and goes with time. In other words, one cannot put one’s life on ´hold´; it is not possible to just step out of the own temporal course of life. Accordingly, Kierkegaard would probably consider it ridiculous to attempt to stop the passing of time, for example by masking aging. Thirdly, the philosopher wrote that death itself has an earnest attitude, as ´its decision´ is always a final one. Whilst the living believe they can always revise, suspend, or postpone decisions, death is radical and non-negotiable. The ´earnest person´ understands that in the moment of death, everything is over. The earnestness of death rouses the living to wakefulness because it signals us that our time is limited and therefore, worthwhile. It also gives us the responsibility of investing our time wisely.

Here, Kierkegaard transforms the negative domination of passing time into something positive by showing that a lifetime is something precious precisely because of its finiteness, and that time, through its transience, gives us the opportunity to determine for ourselves how we want to deal with this rare commodity. What Kierkegaard proposes with his thought on the earnest is a form of *meditiatio mortis*. But his proposal differs from the *meditiatio mortis* tradition of the Baroque period because it is life oriented. His thinking about earnestness is a temporal movement of repetition. This is indeed oriented at the beginning towards the future and its ending - death - but only up to a turning point. After arriving at this turning point, we are to turn our attention back to life, meaning to our present. Anticipating the future here does not mean sorrowfully expecting what the future will bring, and neither is it to be understood as an anticipatory planning for one’s future. Repetition is a dual movement: The goal that it is striving to achieve - an overview of the entirety of one’s own life - will never be reached completely, or once and for all, as this would mean one’s own life has ended. On the contrary, the movement will always refer to the place of its beginning: the present where an individual currently finds themselves. However, the goal is constantly being reached in that it lies in the repetition itself, which is always gaining something new by assimilating the anticipated. Through identifying the dual movement with the successful relationship to the self, Kierkegaard raises the present above the other two aspects of time, the future and the past. He does this because it is only in the present that the synthesis - the dual movement - always returns anew to its beginning and endpoint. When broken down, this means that mentally anticipating one’s own future, which is a confrontation with one’s own ideas of what that future should be and what one would like to look back upon one day, can be helpful in order to craft our own presence accordingly. In doing so, it would certainly make sense to also keep in mind the partially unavailable nature of life. A life can only be good, or well-lived, when its subject neither persists in remaining in the past, nor gets lost in the future, but rather lives in the present that holds both past and future. While the technologies described above assume the individual must struggle against time and its passing to wrest more life from temporality, Kierkegaard is concerned with appropriating the temporality of one’s own life.

## Conclusions

The described suffering from the passing of time is an expression of suffering from the finitude and fugacity of the individual possibilities in a lifetime and the accompanying necessity of making decisions - and when doing so, irreversibly discarding other options.

Regarding the question of how to deal with the temporal structure of one’s own life: What matters is not - or at least not solely - the quantity, but the quality of the own lifetime. One important aspect which causes suffering due to our finite and fugacious lifetime is the fact that time passing by forces people to choose between some life projects and experiences, while consequently having to exclude others. In individualist and consumerist societies more and more people seem to find it difficult to make choices in the face of the unlimited possibilities of self-fulfillment they are confronted with. The necessary condition for being able to make good choices is to have an idea of what is ´good´ or what a good life is. It is a matter of course that especially in pluralistic societies, the question of criteria that can be used to make ´good choice´ in life is difficult to explore, since there are very different ideas of what is good.

Nevertheless, even if the use of techniques like social egg freezing, anti-aging medicine, and physical- and neuro-enhancement can, in some individual cases, free people from the challenges of temporality, this does not necessarily mean that those are good means to achieve a good life. This is because the question of the good life is not dependent on merely expanding a lifetime. That would instead make it possible to postpone the connected challenge of confronting ourselves with the question as to what a good life is repeatedly. There are of course people who are not interested in realizing a good life, and thus are concerned with temporality only under the motto *carpe diem*. For those who just want to ´seize the day´, the use of enhancement techniques might bring the ´benefit´ of gaining more time for experiencing delight. However as already described, people like this should keep in mind that such techniques also contain the risk of undesirable side-effects.

For people, though, who *are* concerned with the question of a good life and aim to live one, it is not possible to avoid the confrontation with their own temporality. Even if this confrontation can be experienced as a painful one, it can also represent an opportunity. When confronted with the necessity of making valuable choices, one must pose the question: What is good, meaningful, and valuable for one´s own life? Temporality does not give an answer, but it gives us the opportunity to pose this question. Confronting oneself with this question is surely not the guarantee for a good life, but it is perhaps the first step to achieving one.

## Data Availability

Not applicable.
